# Panorama of nursing research production in Colombia*

**DOI:** 10.15649/cuidarte.3457

**Published:** 2023-12-19

**Authors:** Ruby Jackeline Rey Triana, Ariel Calderón Ardila, Edgar Francisco Franco Comas, Mayela Nadecza Vega Mendoza, Raquel Rivera Carvajal

**Affiliations:** 1 . Universidad de Santander Facultad de Ciencias Médicas y de la Salud Instituto de Investigación Masira. Bucaramanga, Colombia. E-mail: rub.rey@mail.udes.edu.co Universidad de Santander Universidad de Santander Facultad de Ciencias Médicas y de la Salud Instituto de Investigación Masira Bucaramanga Colombia rub.rey@mail.udes.edu.co; 2 . Universidad de Santander. Facultad de Ciencias Médicas y de la Salud Instituto de Investigación Masira. Bucaramanga, Colombia. E-mail: ar.calderon@mail.udes.edu.co Universidad de Santander Universidad de Santander Facultad de Ciencias Médicas y de la Salud Instituto de Investigación Masira Bucaramanga Colombia ar.calderon@mail.udes.edu.co; 3 .Universidad de Santander. Facultad de Ciencias Médicas y de la Salud Instituto de Investigación Masira. Bucaramanga, Colombia. E-mail: pachitofcomas@hotmail.com Universidad de Santander Universidad de Santander Facultad de Ciencias Médicas y de la Salud Instituto de Investigación Masira Bucaramanga Colombia pachitofcomas@hotmail.com; 4 . Universidad de Santander. Facultad de Ciencias Médicas y de la Salud Instituto de Investigación Masira. Bucaramanga, Colombia. E-mail: mayelavegamendoza59@gmail.com Universidad de Santander Universidad de Santander Facultad de Ciencias Médicas y de la Salud Instituto de Investigación Masira Bucaramanga Colombia mayelavegamendoza59@gmail.com; 5 . Universidad de Santander. Facultad de Ciencias Médicas y de la Salud Instituto de Investigación Masira. Bucaramanga, Colombia. E-mail: raq.rivera@mail.udes.edu.co Universidad de Santander Facultad de Ciencias Médicas y de la Salud Instituto de Investigación Masira Bucaramanga Colombia raq.rivera@mail.udes.edu.co

Research has marked a before and after for nursing, which has made it possible to show care as the nursing discipline's object to the world from its different fields of action and the interdisciplinary work with other professions that complement each other to achieve the goal of promoting human well-being in all its dimensions^1^.

In addition to adhering to a rigorous scientific method, advances in disciplinary knowledge through research development are responsible for disseminating knowledge to the scientific community. That is a contribution to the desired competitiveness in a society, which not only seeks to expand the profession conceptually but also contributes to better professional practice^2^.

As a result of this development, national and international organizations have emerged that, through scientometrics, propose research methods and metrics for journals' classification. International organizations include Clarivate Analytics with Web of Science (WoS) in the United States, Elsevier with Scopus in Amsterdam, and SCImago in Spain^3^^,^^4^.

In Colombia, the Ministry of Science and Technology (MINCIENCIAS), formerly known as COLCIENCIAS, is in charge of promoting and regulating policies related to scientific production, playing various roles ranging from the promotion of research through funding programs and support for research projects to the training of professionals and the creation of scientific collaboration networks. It also promotes the visibility of Colombian research with tools such as the GrupLAC and participation in international scientific networks^5^.

MINCIENCIAS has two applications, CvLAC and GrupLAC, which are part of the SCienTI-Colombia IT platform. The GrupLAC can facilitate identifying, registering, and monitoring research groups in the country ^6^. A research group is defined as a group of people who come together to research and develop knowledge products on one or more subjects^7^. For MINCIENCIAS, it is important to identify the researchers; for this reason, the CvLAC platform stores their CVs and keeps the records and evaluations of their academic and scientific activities - identifying the research areas- and the follow-up of their production^7^.

For a research group to be recognized as such, it must be registered in the GrupLAC system, have at least two members, have at least one year of research experience, be endorsed by at least one institution registered in the InstituLAC, have at least one research project led by a person with a bachelor's degree and a specialist degree, master's, or Ph.D. degree, among other requirements. It is important to note that the affiliation of these groups may be public or private^6^^,^^7^.

In addition to being registered on the CvLAC platform, the researchers who are part of the research groups are included in four categories: researcher, researcher in training, undergraduate student, and affiliated member. These four categories are divided into the following subtypes: emeritus researcher, senior researcher, associate researcher, junior researcher, Ph.D. graduate member, Ph.D. student, master's graduate member, master's student, specialist graduate member, college graduate member, undergraduate student, or affiliated member^8^.

Among the subtypes of researchers the first four are highlighted and described below. The emeritus researcher must have a Ph.D., have completed type-A products, and have completed products as a doctoral director or master's director. The senior researcher must have a Ph.D., produce at least ten type-A products, and training products as director or co-director of four master's theses or a Ph.D. dissertation. The associate researcher must have a Ph.D. or master's degree, three Type-A products, and four new knowledge products in the last five years and have directed or co-directed a Ph.D. dissertation or two master's theses. Finally, the junior researcher must have a Ph.D. or master's degree, a type-A product and four new knowledge products in the last five years^7^.

Considering the requirements that a researcher must fulfill to be part of a group that contributes to the scientific knowledge of our country, it is evident how much time and dedication they have to invest in each of their products, as well as in the orientation of future professionals. They require not only organized teamwork with professional nursing colleagues but also articulation with professionals from different fields to make a quality contribution to nursing and professional practice.

It is in the interest of the Journal's editorial team to identify and recognize the efforts and work of researchers in the field of nursing knowledge. For this purpose, it conducted an analysis of the MINCIENCIAS research groups and a characterization of the researchers from 2017 to 2021.

During this period, 34 groups with nursing topics were identified, 17 of which were endorsed by private institutions and 17 by public institutions. When comparing the groups, no significant differences were observed (p<0.05). Among those categorized in A1, there is one in each group: in the private group, there is the EVEREST nursing research group, and in the public group, there is the Nursing Care for Chronic Patients research group. Category B has a higher percentage of private institutions, and category C has a higher percentage of public institutions. Regarding endorsing institutions, the highest percentage was for educational institutions andone-fifth for healthcare-providing institutions (IPS, for its acronym in Spanish). The number of members and the median number of allocated hours were similar in the two groups, as was the number of articles published. See Table 1.

**Table 1:** Information on nursing research groups in Minciencias

Characteristics	All (34)	Private (17)	Public (17)	P value
GrupLAC category				0.575
A1	5,88(2)	5,88(1)	5.88(1)	
A	23,53(8)	23,58(4)	23.58(4)	
B	35,29(12)	47,06(8)	23.53(4)	
C	32,35(11)	23,53(4)	41.18(7)	
NR	2,94(1)		5.88(1)	
Endorsing institution area				1
Educational	82,35(28)	82,35(14)	82.35(14)	
Healthcare-providing institutions (IPS)	17,65(6)	17,65(3)	17.65(3)	
Number of members				
Median (IQR)	12(9; 18)	11(9; 13)	14(7; 20)	0,5336
Mean ± SD	14,5 ± 11,32	12,35 ± 4,99	16,64 ±	0,2757
			15,16	
Range	(3-68)	(6-25)	(3-68)	
Median allocated hours				
Median (IQR)	6(4; 8)	6(4; 8)	6(4; 7)	0,8838
Scopus				
Number of articles 2017-2021	1128	54,52(615)	52.25(577)	

p value, Qualitative variables Pearson's Chi-Square and Fisher's Exact Chi-Square tests.Quantitative variables Student's t-test and Mann-Whitney U test.

In the 34 groups, 1287 researchers were identified. Of these, 21.53% (471) are active, 52.86% (249) of them are affiliated to research groups sponsored by public institutions, and the remaining 47.13% (222) to private ones. According to the categorization in the CvLAC, there is a tendency for higher percentages of those affiliated to private institutions in the category of associate researchers, with 14.21% (32/222), and in public institutions with 6.45% (16). The hours allocated to the research group were similar, with a median of 6 hours (private institutions) and 5 hours (public institutions) per week. Regarding the level of education, it was found that public institutions have a higher percentage of Ph.D. graduates than private institutions, the percentage of master's graduates is higher in private institutions, and the percentage of researchers with postgraduate studies in nursing is higher in public institutions. In comparison, the percentage is higher in private institutions in epidemiology and education. See Table 2.

Intellectual production (articles, book chapters) was found to have similar medians; the Scopus h-index was calculated in 220 researchers, of which the median for private institutions' researchers was 4 and 5 for public institutions' researchers, with no differences between groups. According to the publications, the most common research topics were public health, followed by teaching, and chronic NCDs (non-communicable diseases) and students in third place. See Table 2.

**Table 2:** Characteristics of nursing researchers in Minciencias %(n)

Characteristics	All (471)	Private (222)	Public (249)	P value
CvLAC category				0,054
Emeritus researcher	0,85(4)	0,90(2)	0,81(2)	
Senior researcher	6,38(30)	6,31(14)	6,45(16)	
Associate researcher	10,21(48)	14,21(32)	6,45(16)	
Junior researcher	22,55(106)	22,97(51)	22,18(55)	
NR	60,00(282)	55,41(23)	64,11(159)	
Allocated hours. Median (IQR)	6(4; 10)	6(4; 10)	5(4; 10)	0,6792
Education level				< 0,001
Ph.D. degree	33,33(157)	27,03(60)	38,96(97)	
Master's degree	46,28(218)	52,70(117)	40,56(101)	
Specialist degree	7,01(33)	11,71(26)	2,81(7)	
Bachelor's degree	4,67(22)	4,95(11)	4,42(11)	
NR	8,70(41)	3,60(8)	13,25(33)	
Postgraduate fields of study				< 0,001
Nursing	37,37(176)	29,28(65)	44,58(111)	
Education	12,53(59)	15,32(34)	10,04(25)	
Epidemiology	6,79(32)	10,81(24)	3,21(8)	
Healthcare management and public health	6,16(29)	7,21(16)	5,22(3)	
Psychology	1,70(8)	2,25(5)	1,20(3)	
Others	26,75(126)	31,53(70)	22,49(56)	
NR	8,70(41)	3,60(8)	13,25(33)	
Endorsing institution area				0,243
Educational	85,77(404)	83,78(186)	87,55(218)	
Healthcare-providing institutions (IPS)	14,23(67)	16,22(36)	12,45(31)	
Number of articles. Median (IQR)	8(3; 18)	8(3; 16,5)	9(3; 19)	0,4008
Scopus h-index. Median (IQR) (207)	4(2; 7)	4(2; 7)	5(2; 7)	0,4072
Topics of publication				
Public health	29,72(140)	31,08(69)	28,51(71)	0,306
Teaching	26,54(125)	26,58(59)	26,51(66)	0,986
Chronic NCDs	22,72(107)	22,97(51)	22,49(56)	0,901
Woman	20,59(97)	22,07(49)	19,48(48)	0,454
Psychology	20,59(97)	22,52(50)	18,88(47)	0,329
Adolescents	18,26(86)	20,72(46)	16,06(40)	0,192
Inpatients	18,05(85)	17,12(38)	18,88(47)	0,620
Occupational health	17,62(83)	18,02(40)	17,27(43)	0,831
Geriatrics	17,20(81)	18,47(41)	16,06(40)	0,490
ICU	16,14(76)	12,61(28)	19,28(48)	0,050
Sexual health	13,85(64)	14,41(32)	12,85(32)	0,621
Pediatrics	13,59(64)	13,51(30)	13,65(34)	0,964
Newborns	8,70(41)	7,66(17)	9,64(24)	0,447
COVID-19	7,64(36)	7,21(16)	8,03(20)	0,737

p value Qualitative variables Pearson's Chi-Square and Fisher's Exact Chi-Square tests. Quantitative variables Student's t-test and Mann-Whitney U test. Abbreviations: NR: Not reported. IPS: Healthcare providing institutions. ICU: Intensive care unit. IQR: Interquartile range.

It is evident that researchers in both the public and private sectors devote additional time to developing products that contribute to the scientific community, in addition to their roles as professional nurses. According to the analysis, the percentage of active researchers is considerably low; in this regard, greater benefits and incentives could be considered to encourage nurse researchers to achieve greater scientific production in this field of knowledge. Similarly, healthcare-providing institutions (IPS), although fewer in number than educational institutions, have made efforts to grow and contribute to the field of research, as shown in Table 1. Institutions, in general, are increasingly interested in having within their research groups professionals with a high level of education, such as master's and Ph.D. graduates in nursing or related fields, who will contribute not only to the growth of the profession but also to the provision of evidence-based care, with the use of nursing theories and theoretical models that support efficiency and effectiveness in prevention and recovery in healthcare.

Researchers in the public and private sectors, continue to develop research and publications in nursing despite new challenges such as the demands of educational qualifications, ongoing publications, advising and mentoring doctoral or master's students, and other additional functions apart to nursing practice. The development of research in the health scientific community, while showing an upward trend, has been slow compared to the growth of researchers and scientific production in other Latin American countries^9^.

The invitation is extended to active researchers, those who have not continued to publish for various reasons, and new researchers to continue devoting part of their time to the demanding but rewarding field of research. They are invited to work hand in hand with other professionals to strengthen interdisciplinary work and enrich the field of nursing knowledge with new ideas, alternatives, and growth options for the profession and professional practice.
