# Supplementary material for: Panorama of nursing research production in Colombia
Source: Rev Cuid. 2023 Dec 19;14(3):e01. doi: 10.15649/cuidarte.3457 (PMC11560090; doi:10.15649/cuidarte.3457)
Supplement: Translation

# Translation: Panorama da produção de pesquisa em enfermagem na Colômbia

A pesquisa marcou um antes e um depois para a enfermagem, o que permitiu ao mundo mostrar o cuidado como objeto da disciplina a partir dos diferentes campos de atuaáo da profissáo, bem como o trabalho interdisciplinar com outras profissoes que se complementam, para alcanzar o objetivo cujo objetivo é promover o bem-estar do ser humano em todas as suas dimensoes^1^.

Os avanzos no conhecimento disciplinar por meio do desenvolvimento de pesquisas, além de obedecerem a um rigoroso método científico, sao responsáveis pela disseminaáo do conhecimento a comunidade científica, o que representa uma contribuido para a desejada competitividade na sociedade, que busca náo apenas a expansáo conceitual da profissáo, mas sim contribuir para um melhor desempenho na prática profissional^2^.

Consequentemente, com este desenvolvimento, surgiram organizares a nível nacional e internacional que, através do campo da cienciometria, propoem métodos de investigado e métricas para a classificaáo de revistas. Entre as organizares internacionais estáo a Clarivate Analytics com base Web of Science (WoS) nos Estados Unidos, a Elsevier com Scopus em Amsterdá e a SCImago na Espanha^3^^,^^4^.

Na Colombia, o Ministério da Ciencia e Tecnología (MINCIENCIAS), anteriormente conhecido como COLCIENCIAS, é responsável por promover e regular as políticas relacionadas a produáo científica, desempenhando diversas funóes que váo desde a promoáo da pesquisa, passando por programas de financiamento e apoio a projetos de pesquisa, até a formaáo de profissionais e a criaáo de redes de colaborado científica; Da mesma forma, promove a visibilidade da pesquisa colombiana através de ferramentas como o GrupLAC e a participado em redes científicas internacionais^5^.

MINCIENCIAS possui duas aplicaóes, CvLAC e GrupLAC que fazem parte da plataforma informática SCienTI- Colombia. Dentro do GrupLAC pode ser facilitada a identificado, registro e monitoramento de grupos de pesquisa no país^6^. Um grupo de pesquisa é definido como um grupo de pessoas que se reúnem para investigar e gerar produtos de conhecimento sobre um ou mais tópicos^7^. Para o MINCIENCIAS é importante a identificado dos pesquisadores, por isso, junto a plataforma CvLAC sáo registrados seus currículos, o registro e a avaliado das atividades académicas e científicas, identificando as áreas de pesquisa e monitorando sua produdo.

Para que um grupo de pesquisa seja reconhecido como tal, deve estar cadastrado no sistema GrupLAC, ter pelo menos dois membros, um ano ou mais de experiencia em pesquisa, ser endossado por pelo menos uma instituido cadastrada no InstituLAC, ter pelo menos um projeto pesquisas com líder que possua graduado e especializado, mestrado ou doutorado, entre outros. Ressalta-se que esses grupos podem ser públicos ou privados.

Os pesquisadores que integram os grupos de pesquisa, além de estarem cadastrados na plataforma CvLAC, estáo vinculados dentro de 4 categorias: pesquisador, pesquisador em formado, estudante de graduado e membro vinculado; Essas 4 categorias sáo subdivididas em subtipos: pesquisador emérito, pesquisador senior, pesquisador associado, pesquisador júnior, membro vinculado ao doutorado, doutorando, membro vinculado ao mestrado, mestrando, membro com especializado, membro com graduado, estudante de graduado ou vinculado membro^8^.

Dentro dos subtipos de pesquisadores, destacam-se os 4 primeiros, descritos a seguir: o pesquisador emérito que deve possuir doutorado, possuir produtos tipo A, além de produtos de formado como diretor de doutorado concluído ou diretor de mestrado. Por outro lado, existe o investigador sénior que deve ter doutoramento, produdo mínima de 10 produtos tipo A, bem como produtos de formado como diretor ou codiretor de 4 projetos de mestrado ou de tese de doutoramento. O investigador associado deverá ter doutoramento ou mestrado concluído, 3 produtos tipo A e 4 novos produtos de conhecimento nos últimos 5 anos, ter dirigido ou codirigido uma tese de doutoramento ou 2 dissertaóes de mestrado; por fim, o pesquisador júnior que deverá possuir doutorado ou mestrado, 1 produto tipo A e 4 produtos nos últimos 5 anos^7^.

Tendo em conta os requisitos que um investigador deve cumprir para fazer parte de um grupo que contribui para o conhecimento científico no nosso país, fica evidente o tempo e a dedicado que deve entregar em cada um dos seus produtos, bem como na orientado dos futuros profissionais. Exigem um trabalho organizado e em equipe náo só com os colegas profissionais de enfermagem, mas também coordenado com profissionais de diversas áreas, a fim de dar uma contribuido de qualidade a profissáo e ao exercício profissional.

É do interesse da equipa editorial da Revista identificar e reconhecer o esforo e trabalho dos investigadores na área do conhecimento da enfermagem, para o efeito foi realizada uma análise dos grupos de investigado do MINCIENCIAS, bem como uma caracterizado dos pesquisadores no período de 2017 a 2021.

Neste período foram identificados 34 grupos com temática de Enfermagem, sendo 17 referendados por instituióes privadas e 17 por instituióes públicas. Na comparacáo dos grupos nao foram observadas diferenas significativas (p<0,05), dentro daquelas categorizadas em A1 Há um em cada grupo, no grupo privado há o grupo de pesquisa em enfermagem EVEREST e no público há o grupo de assistencia de enfermagem a pacientes crónicos; em B verifica-se uma porcentagem mais elevada nas instituyes privadas e na categorizaáo C verifica-se uma porcentagem mais elevada nas instituyes públicas. Em relaáo a instituido que aprova, a porcentagem mais elevada foi das entidades educativas e das Instituyes Prestadoras de Saúde (IPS) num quinto. O número de membros e a mediana de horas de dedicado foram semelhantes nos dois grupos, assim como o número de artigos publicados. Consulte a Tabela 1.

**Tabela 1:** Informales sobre grupos de pesquisa com temas de Enfermagem em Minciencias

Características	Todos (34)	Privado (17)	Público (17)	Valor p
Categoria Gruplac				0.575
A1	5,88(2)	5,88(1)	5,88(1)	
A	23,53(8)	23,58(4)	23,58(4)	
B	35,29(12)	47,06(8)	23,53(4)	
C	32,35(11)	23,53(4)	41,18(7)	
NR	2,94(1)		5,88(1)	
Instituido de apoio da área				1
Educativo	82,35(28)	82,35(14)	82,35(14)	
IPS	17,65(6)	17,65(3)	17,65(3)	
Número de integrantes				
Médio (RI)	12(9; 18)	11(9; 13)	14(7; 20)	0,5336
Média ± DE	14,5 ± 11,32	12,35 ± 4,99	16,64 ± 15,16	0,2757
Faixa	(3-68)	(6-25)	(3-68)	
Média de horas de dedicado				
Médio (RI)	6(4; 8)	6(4; 8)	6(4; 7)	0,8838
Scopus				
Número artigos 2017-2021	1128	54,52(615)	52,25(577)	

Valor de P Variáveis qualitativas Testes Qui-Quadrado de Pearson e Exato de Fisher. Variáveis numéricas Teste T-Student e Mann Whitney U.

Nos 34 grupos foram identificados 1.287 pesquisadores e destes 21,53% (471) atuavam, dentre os quais 52,86% (249) estavam vinculados a grupos de pesquisa patrocinados por instituyes públicas e os restantes 47,13% (222) a privadas. De acordo com a categorizaáo na CvLAC, há uma tendencia de maiores percentuais nos vinculados a instituyes privadas para pesquisadores associados, onde 14,21% (32/222) e nas instituyes públicas representam 6,45% (16); as horas de dedicado foram semelhantes com mediana de 6 e 5 horas semanais. Quanto ao nivel de escolaridade, identificou-se que com o doutorado nas instituyes públicas eles tem um percentual maior do que nas privadas, com o mestrado é maior nas privadas e a área de pós- graduado em relaáo a Enfermagem teve um percentual maior nas públicas, enquanto na epidemiologia e na educaáo o percentual é maior nas privadas. Consulte a Tabela 2.

A produáo intelectual (artigos, capítulos de livros) foi encontrada com mediana semelhante, o índice Scopus H foi identificado para 220 pesquisadores, dos quais a mediana em pesquisadores de instituyes privadas foi 4 e em instituyes públicas foi 5, sem diferenas nos grupos. Os temas dos pesquisadores segundo as publicares mais prevalentes foram saúde pública, seguido do ensino, em terceiro lugar, DCNT (Doena Coronariana Náo Transmissível) e estudantes. Ver Tabela 2.

**Tabela 2:** Caracterizado dos Pesquisadores com temas de Enfermagem em Minciencias. %(n).

Características	Todos (471)	Privado (222)	Público (249)	Valor p
Categorizaao Cvlac				0,054
Investigador Emérito	0,85(4)	0,90(2)	0,81(2)	
Investigador Sénior	6,38(30)	6,31(14)	6,45(16)	
Investigador Associado	10,21(48)	14,21(32)	6,45(16)	
Investigador Junior	22,55(106)	22,97(51)	22,18(55)	
NR	60,00(282)	55,41(23)	64,11(159)	
Horas de dedicado. Médio (RI)	6(4; 10)	6(4; 10)	5(4; 10)	0,6792
Nível educativo				< 0,001
Doutorado	33,33(157)	27,03(60)	38,96(97)	
Mestrado - Magister	46,28(218)	52,70(117)	40,56(101)	
Especializado	7,01(33)	11,71(26)	2,81(7)	
Graduado	4,67(22)	4,95(11)	4,42(11)	
NR	8,70(41)	3,60(8)	13,25(33)	
Área da pós-graduaao				< 0,001
Enfermagem	37,37(176)	29,28(65)	44,58(111)	
Educado	12,53(59)	15,32(34)	10,04(25)	
Epidemiologia	6,79(32)	10,81(24)	3,21(8)	
Administrado em Saúde- SP	6,16(29)	7,21(16)	5,22(3)	
Psicologia	1,70(8)	2,25(5)	1,20(3)	
Outros	26,75(126)	31,53(70)	22,49(56)	
NR	8,70(41)	3,60(8)	13,25(33)	
Instituido de apoio da área				0,243
Educativo	85,77(404)	83,78(186)	87,55(218)	
IPS	14,23(67)	16,22(36)	12,45(31)	
Número de artigos. Médio (RI)	8(3; 18)	8(3; 16.5)	9(3; 19)	0,4008
Índice H. Scopus. Médio (RI) (207)	4(2; 7)	4(2; 7)	5(2; 7)	0,4072
Temáticas das publicaoes				
Saúde Pública	29,72(140)	31,08(69)	28,51(71)	0,306
Docéncia	26,54(125)	26,58(59)	26,51(66)	0,986
ECNT	22,72(107)	22,97(51)	22,49(56)	0,901
Mulher	20,59(97)	22,07(49)	19,48(48)	0,454
Psicologia	20,59(97)	22,52(50)	18,88(47)	0,329
Adolescentes	18,26(86)	20,72(46)	16,06(40)	0,192
Hospitalizados	18,05(85)	17,12(38)	18,88(47)	0,620
Saúde Ocupacional	17,62(83)	18,02(40)	17,27(43)	0,831
Geriatria	17,20(81)	18,47(41)	16,06(40)	0,490
UTI	16,14(76)	12,61(28)	19,28(48)	0,050
Saúde Sexual	13,85(64)	14,41(32)	12,85(32)	0,621
Pediatria	13,59(64)	13,51(30)	13,65(34)	0,964
Recém-nascido	8,70(41)	7,66(17)	9,64(24)	0,447
Covid-19	7,64(36)	7,21(16)	8,03(20)	0,737

Valor de P Variáveis qualitativas Testes Qui-Quadrado de Pearson e Exato de Fisher, Variáveis numéricas Teste U de Mann-Whitney. Abreviaturas: NR: Sem

Relatório. IPS: Instituido Prestadora de Saúde. UTI: Unidade de Terapia Intensiva. IR: Intervalo Interquartil.

É evidente que pesquisadores dos setores público e privado dedicam tempo adicional as funóes que desempenham como profissionais de enfermagem, no desenvolvimento de produtos com os quais contribuem para a comunidade científica. De acordo com a análise efetuada, verifica-se que a percentagem de investigadores ativos é consideravelmente baixa; nesse sentido, poderiam ser considerados maiores benefícios e estímulos para incentivar os profissionais de enfermagem que se dedicam a pesquisa, a fim de alcanarem maior produáo científica nesta área do conhecimento. Da mesma forma, as instituióes prestadoras de servidos de saúde (IPS), apesar de serem em número menor se comparadas as instituióes de ensino, tem se esforzado para crescer e contribuir com o campo da pesquisa como se ve na Tabela 1.

As instituióes em geral estáo cada vez mais preocupadas em ter dentro de seus grupos de pesquisa, profissionais com alto nivel de formaáo como mestres e doutores em enfermagem ou áreas afins que contribuam náo só para o crescimento da profissáo, mas também para a prestado de cuidados baseados em evidencias, com a utilizado de teorias e modelos teóricos de enfermagem que apoiam a eficiencia e a eficácia na prevengo e recuperado no ámbito dos cuidados de saúde.

Investigadores dos setores público e privado continuam a desenvolver investigado e publicaóes na área da enfermagem apesar dos novos desafios como exigencias de formado académica, publicaóes permanentes, aconselhamento e apoio a alunos de doutoramento ou mestrado, e fundes adicionais na área. profissáo, entre outros. O desenvolvimento das pesquisas na comunidade científica em saúde, embora apresentando crescimento ascendente, tem ocorrido de forma lenta, se comparado ao crescimento dos pesquisadores e da produdo científica em outros países latino-americanos.

O convite é extensivo aos investigadores ativos, aos que por motivos diversos náo deram continuidade as suas publicades e aos novos investigadores, para que continuem a dedicar parte do seu tempo a este campo táo exigente, mas táo gratificante como a investigado, para trabalharem a máo de outros profissionais. que permitam fortalecer o trabalho interdisciplinar e enriquecer o campo do conhecimento da enfermagem com novas ideias, alternativas e opóes de crescimento para a profissáo e para o exercício do exercício profissional.

# Translation: Panorama de la producción investigativa de enfermería en Colombia

La investigación ha marcado un antes y un después para la enfermería, lo que ha permitido mostrar al mundo el cuidado como objeto de la disciplina desde los diferentes campos de desempeño de la profesión, así como el trabajo interdisciplinario con otras profesiones que se complementan, para lograr el objetivo cuya meta es promover el bienestar del ser humano en todas sus dimensiones^1^.

Los avances en el conocimiento disciplinar a través del desarrollo investigativo además de cumplir con un método científico riguroso, se hace cargo de la difusión del conocimiento a la comunidad científica, lo que representa un aporte a la competitividad deseada en la sociedad, que busca no solo la ampliación conceptual de la profesión, sino contribuir con un mejor ejercicio en la práctica profesional^2^.

En consecuencia, a este desarrollo han surgido organizaciones a nivel nacional e internacional, que, a través del campo de la cienciometría proponen métodos para la investigación y métricas para la clasificación de las revistas. Dentro de las organizaciones internacionales se encuentran Clarivate Analytics con la base Web of Sciencie (WoS) de Estados Unidos, Elsevier con Scopus en Ámsterdam y SCImago de España^3^^,^^4^.

En Colombia el Ministerio de Ciencia Teconología e Innovación (MINCIENCIAS), anteriormente conocido como COLCIENCIAS, es el encargado de promover y regular las políticas relacionadas con la producción científica, desempeñando diversos roles que van desde la promoción de la investigación a través de programas de financiamiento y apoyo a proyectos de investigación, hasta la formación de profesionales y la creación de redes de colaboración científica; de igual forma promueve la visibilidad de la investigación colombiana a través de herramientas como el GrupLAC y la participación en redes científicas internacionales^5^.

MINCIENCIAS cuenta con dos aplicativos, CvLAC y GrupLAC que hacen parte de la plataforma informática SCienTI-Colombia. Dentro de GrupLAC se puede facilitar la identificación, el registro y seguimiento de los grupos de investigación en el país^6^. Un grupo de investigación se define como un conjunto de personas que se unen para investigar y generar productos de conocimiento en uno o varios temas^7^. Para MINCIENCIAS es importante tener identificados a los investigadores, por este motivo, con la plataforma CvLAC se tiene inscrita su hoja de vida, el registro y evaluación de las actividades académicas y científicas, identificando las áreas de investigación y el seguimiento de su producción^7^.

Para que un grupo de investigación sea reconocido como tal, debe encontrarse registrado en el sistema GrupLAC, tener como mínimo dos integrantes, un año o más de experiencia investigativa, estar avalado por al menos una institución registrada en el InstituLAC, tener al menos un proyecto de investigación con un líder que posea título de pregrado y un título de especialización, maestría o doctorado entre otros. Cabe resaltar que estos grupos pueden ser de filiación pública o privada^6^^,^^7^.

Los investigadores que hacen parte de los grupos de investigación, además de estar registrados en la plataforma de CvLAC son vinculados dentro de 4 categorías: investigador, investigador en formación, estudiante de pregrado e integrante vinculado; estas 4 categorías se subdividen en subtipos: investigador emérito, investigador sénior, Investigador asociado, investigador junior, integrante vinculado con doctorado, estudiante de doctorado, integrante vinculado con maestría, estudiante de maestría, integrante con especialización, integrante con pregrado, estudiante de pregrado o integrante vinculado^8^.

Dentro de los subtipos de investigadores destacan los 4 primeros que se describen a continuación: el investigador emérito quien debe contar con doctorado, tener productos tipo A, así como productos de formación como director de doctorado finalizado o director de maestría. Por otra parte, está el investigador senior quien debe contar con doctorado, una producción mínima de 10 productos tipo A, así como productos de formación como director o codirector de 4 trabajos de maestría o una tesis de doctorado. El investigador asociado debe tener doctorado o maestría finalizada, 3 productos tipo A y 4 productos de nuevo conocimiento en los últimos 5 años, haber dirigido o codirigido una tesis de doctorado o 2 trabajos de maestría; por último, el investigador junior quien debe contar con título de doctorado o maestría, 1 producto tipo A y 4 productos en los últimos 5 años^7^.

Teniendo en cuenta los requisitos que debe cumplir un investigador para poder hacer parte de un grupo que aporte al conocimiento científico en nuestro país, se hace evidente el tiempo y la dedicación que estos deben entregar en cada uno de sus productos, así como en la orientación de futuros profesionales. Requieren de un trabajo organizado y en equipo no solo con los colegas profesionales de enfermería, sino de la articulación con profesionales de distintas áreas, con el fin de realizar un aporte de calidad a la profesión y al ejercicio profesional.

Es del interés del equipo editorial de la Revista identificar y reconocer el esfuerzo y trabajo de los investigadores en el campo de conocimiento de enfermería, para ello realizó un análisis de los grupos de investigación en MINCIENCIAS, así como una caracterización de los investigadores en el periodo de 2017 a 2021.

En este periodo de tiempo se identificaron 34 grupos con temática de Enfermería, dentro de los cuales 17 están avalados por instituciones privadas y 17 por públicas, al comparar los grupos no se aprecian diferencias significativas (p<0.05), dentro de los categorizados en A1 hay uno en cada grupo, en el privado está el grupo de investigación de enfermería EVEREST y en el público el grupo cuidado de enfermería al paciente crónico; en B se aprecia un porcentaje mayor en las instituciones privadas y en categorización C un mayor porcentaje en instituciones públicas. En relación con la institución que avala el mayor porcentaje fue por entidades de educación y por Instituciones Prestadoras de Salud (IPS) en una quinta parte. El número de integrantes y la mediana de horas de dedicación fueron similares en los dos grupos, así como el número de artículos publicados. Ver Tabla 1.

Tabla 1. Información grupos de Investigación con temática de Enfermería en Minciencias

**Table:** 

Características	Todos (34)	Privado (17)	Público (17)	Valor p
Categoría Gruplac				0,575
A1	5,88(2)	5,88(1)	5,88(1)	
A	23,53(8)	23,58(4)	23,58(4)	
B	35,29(12)	47,06(8)	23,53(4)	
C	32,35(11)	23,53(4)	41,18(7)	
NR	2,94(1)		5,88(1)	
Área Institución avaladora				1
Educativo	82,35(28)	82,35(14)	82,35(14)	
IPS	17,65(6)	17,65(3)	17,65(3)	
Número de integrantes				
Mediana(RI)	12(9; 18)	11(9; 13)	14(7; 20)	0,5336
Promedio ± DE	14,5 ± 11,32	12,35 ± 4,99	16,64 ± 15,16	0,2757
Rango	(3-68)	(6-25)	(3-68)	
Mediana horas de dedicación				
Mediana (RI)	6(4; 8)	6(4; 8)	6(4; 7)	0,8838
Scopus				
Numero artículos 2017-2021	1128	54,52(615)	52,25(577)	

Valor p Variables cualitativas prueba Chi Cuadrado de Pearson y Exacta de Fisher. Variables numéricas prueba T-Student y U de Mann Whitney.

En los 34 grupos se identificaron 1287 investigadores y de estos el 21,53% (471) activos, dentro de los cuales el 52,86% (249) vinculados a grupos de investigación patrocinados por instituciones públicas y el restante 47,13% (222) a privadas. Según la categorización en el CvLAC se aprecia tendencia a porcentajes mayores en los vinculados a instituciones privadas para investigadores, asociado, donde el 14,21% (32/222) y en las públicas representan el 6,45% (16); las horas de dedicación fueron similares con mediana de 6 y 5 horas semanales. Respecto del nivel educativo se identificó que con doctorado en las instituciones públicas tienen un mayor porcentaje que las privadas, con maestría es mayor en las privadas y el área de posgrado con relación a Enfermería se parecía mayor porcentaje en las públicas, mientras que en epidemiología y educación el porcentaje es mayor en las privadas. Ver Tabla 2.

La producción intelectual (artículos, capítulos de libro) se encontró con mediana similar, el índice H de Scopus fue identificado para 220 investigadores, de los cuales la mediana en los investigadores de instituciones privadas fue de 4 y en los públicos de 5, sin diferencias en los grupos. Las temáticas de los investigadores según las publicaciones con mayor prevalencia fueron salud pública, seguidamente docencia, en tercer lugar, ECNT (Enfermedad Coronaria no Transmisibles y estudiantes). Ver Tabla 2.

**Tabla 2:** Caracterización de los Investigadores con temática de Enfermería en Minciencias %(n)

Características	Todos (471)	Privado (222)	Público (249)	Valor p
Categorización Cvlac				0,054
Investigador Emérito	0,85(4)	0,90(2)	0,81(2)	
Investigador Senior	6,38(30)	6,31(14)	6,45(16)	
Investigador Asociado	10,21(48)	14,21(32)	6,45(16)	
Investigador Junior	22,55(106)	22,97(51)	22,18(55)	
NR	60,00(282)	55,41(23)	64,11(159)	
Horas de dedicación. Mediana (RI)	6(4; 10)	6(4; 10)	5(4; 10)	0,6792
Nivel educativo				< 0,001
Doctorado	33,33(157)	27,03(60)	38,96(97)	
Maestría - Magister	46,28(218)	52,70(117)	40,56(101)	
Especialización	7,01(33)	11,71(26)	2,81(7)	
Pregrado	4,67(22)	4,95(11)	4,42(11)	
NR	8,70(41)	3,60(8)	13,25(33)	
Área del posgrado				< 0,001
Enfermería	37,37(176)	29,28(65)	44,58(111)	
Educación	12,53(59)	15,32(34)	10,04(25)	
Epidemiologia	6,79(32)	10,81(24)	3,21(8)	
Administración en Salud- SP	6,16(29)	7,21(16)	5,22(3)	
Psicología	1,70(8)	2,25(5)	1,20(3)	
Otros	26,75(126)	31,53(70)	22,49(56)	
NR	8,70(41)	3,60(8)	13,25(33)	
Área Institución avaladora				0,243
Educativo	85,77(404)	83,78(186)	87,55(218)	
IPS	14,23(67)	16,22(36)	12,45(31)	
Número de artículos. Mediana(RI)	8(3; 18)	8(3; 16,5)	9(3; 19)	0,4008
Índice H. Scopus. Mediana(RI) (207)	4(2; 7)	4(2; 7)	5(2; 7)	0,4072
Temáticas de las publicaciones				
Salud Publica	29,72(140)	31,08(69)	28,51(71)	0,306
Docencia	26,54(125)	26,58(59)	26,51(66)	0,986
ECNT	22,72(107)	22,97(51)	22,49(56)	0,901
Mujer	20,59(97)	22,07(49)	19,48(48)	0,454
Psicología	20,59(97)	22,52(50)	18,88(47)	0,329
Adolescentes	18,26(86)	20,72(46)	16,06(40)	0,192
Hospitalizados	18,05(85)	17,12(38)	18,88(47)	0,620
Salud Ocupacional	17,62(83)	18,02(40)	17,27(43)	0,831
Geriatría	17,20(81)	18,47(41)	16,06(40)	0,490
UCI	16,14(76)	12,61(28)	19,28(48)	0,050
Salud Sexual	13,85(64)	14,41(32)	12,85(32)	0,621
Pediatría	13,59(64)	13,51(30)	13,65(34)	0,964
Recién Nacido	8,70(41)	7,66(17)	9,64(24)	0,447
Covid-19	7,64(36)	7,21(16)	8,03(20)	0,737

Valor p Variables cualitativas Prueba Chi Cuadrado de Pearson y Exacta de Fisher, Variables numéricas Prueba U de Mann-Whitney. Siglas: NR: No Reporte. IPS: Institución Prestadora de Salud. UCI: Unidad de Cuidados Intensivos. RI: Rango Intercuartílico.

Se hace evidente que los investigadores desde el sector público y privado dedican tiempo adicional a las funciones que desempeñan como profesionales de enfermería, en el desarrollo de productos que aportan a la comunidad científica. De acuerdo con el análisis realizado se encuentra que el porcentaje de investigadores activos es considerablemente bajo; al respecto se podrían considerar mayores beneficios y estímulos para incentivar en los profesionales de enfermería que se dedican a investigar, con el fin de lograr mayor producción científica en este campo de conocimiento. De igual forma las instituciones prestadoras de servicios de salud (IPS) a pesar de ser un número menor comparado con las instituciones educativas, han ido realizando esfuerzos en crecer y aportar al campo de la investigación como se observa en la Tabla 1.

Las instituciones en general se preocupan cada día más por tener dentro de sus grupos de investigación, profesionales con alto nivel de formación como magíster y doctores en enfermería o en áreas afines que aporten no solo al crecimiento de la profesión, sino a brindar un cuidado basado en la evidencia, con el uso de las teorías y modelos teóricos de enfermería que respaldan la eficiencia y efectividad en la prevención y recuperación en el marco de la atención de la salud.

Los investigadores de los sectores público y privado, continúan desarrollando investigaciones y publicaciones en el campo de la enfermería pese a los nuevos desafíos como las exigencias en torno a la formación académica, publicaciones permanentes, asesoría y acompañamiento de estudiantes de doctorado o maestría y funciones adicionales al ejercicio de su profesión, entre otros. Los desarrollos investigativos en la comunidad científica de la salud, aunque presentan un crecimiento ascendente se ha dado de forma lenta, comparado con el crecimiento de investigadores y de producción científica en los demás países latinoamericanos^9^.

Se extiende la invitación a los investigadores activos, a los que por distintos motivos no han continuado con sus publicaciones y a los nuevos investigadores, a seguir dedicando parte de su tiempo a este campo tan exigente pero tan gratificante como lo es la investigación, a trabajar de la mano de otros profesionales que permitan fortalecer el trabajo interdisciplinar y enriquecer el campo de conocimiento de la enfermería con nuevas ideas, alternativas y opciones de crecimiento para la profesión y la práctica del ejercicio profesional.
